# A Tiny Heart, a Massive Challenge: Case Presentation of Pericardial Effusion and Cardiac Tamponade in an Extremely Preterm Neonate and Corresponding Literature Review

**DOI:** 10.7759/cureus.114448

**Published:** 2026-08-12

**Authors:** Sachin Sakharkar, Deepika Tiwari, Srinivas Lakshmivenkateshiah, Rashmi Yemul

**Affiliations:** 1 Neonatology, KIMS Hospitals, Thane, IND; 2 Neonatology, KIMS Hospital, Thane, IND; 3 Pediatric Cardiology, KIMS Hospitals, Thane, IND

**Keywords:** extremely low birth weight, extreme prematurity, pericardial effusion, pericardiocentesis, persistent pulmonary hypertension, picc line

## Abstract

Massive pericardial effusion requiring pericardiocentesis in an extremely preterm, extremely low birth weight (ELBW) neonate is exceedingly rare and infrequently described in neonatal literature.

We report the successful management and favorable neurodevelopmental outcome of a 24-week gestation male baby (birth weight 640 g) who developed hemodynamically significant, massive pericardial effusion during a neonatal intensive care unit course. The infant’s clinical course was complicated by severe respiratory distress syndrome, persistent pulmonary hypertension of the newborn (PPHN), prolonged ventilator dependence, feed intolerance, and the requirement of a peripherally inserted central catheter (PICC) for total parenteral nutrition (TPN) administration. On day 21 of life, the infant had clinical deterioration with features of circulatory shock and worsening respiratory status. Serial chest radiographs demonstrated progressive cardiomegaly with a characteristic “water-bottle” cardiac silhouette, suggestive of significant pericardial effusion. Echocardiographic evaluation confirmed massive pericardial effusion with cardiorespiratory compromise. The PICC line was immediately removed, and emergency pericardiocentesis was performed. Performing an invasive pericardial drainage procedure in a neonate at the threshold of viability posed considerable technical and hemodynamic challenges. Following the intervention, the infant required respiratory support, antimicrobial therapy, nutritional rehabilitation, and multidisciplinary care in a tertiary neonatal intensive care unit. Gradual cardiopulmonary stabilization was achieved, leading to successful discharge. At follow-up, infant demonstrated satisfactory linear growth with no evidence of cognitive, auditory, or visual impairment.

This case highlights the rarity, diagnostic complexity, and therapeutic challenges of massive pericardial effusion in ELBW neonates, and emphasizes the importance of early radiological recognition, prompt echocardiographic evaluation, timely intervention, and multidisciplinary management to improve survival and long-term outcomes in this highly vulnerable population.

## Introduction

Pericardial effusion in neonates is an uncommon but potentially life-threatening complication that may rapidly progress to cardiac tamponade, hemodynamic compromise, and cardiovascular collapse if not recognized and treated promptly. Although the overall incidence is low, catheter-associated pericardial effusion has emerged as an important acquired cause of neonatal cardiac tamponade in contemporary neonatal intensive care units, particularly with the widespread use of umbilical venous catheters (UVCs) and peripherally inserted central catheters (PICCs) for prolonged administration of parenteral nutrition, medications, and vasoactive agents [1,2]. The reported incidence of catheter-associated pericardial effusion in neonates ranges from 0.07% to 2%, with mortality being particularly high when tamponade is associated with delayed recognition or failure to achieve timely drainage [1,2].

Extremely preterm and extremely low birth weight (ELBW) infants may be particularly vulnerable to the hemodynamic consequences of pericardial fluid accumulation. Their small cardiac chambers, immature myocardium, thin atrial walls, and limited intrathoracic and pericardial reserve provide little physiological tolerance to external cardiac compression. In addition, these infants frequently require prolonged central venous access, increasing their exposure to catheter-related complications [1,3]. Consequently, even a relatively small pericardial effusion may substantially impair cardiac filling and systemic perfusion. The clinical presentation may be nonspecific and can mimic sepsis, worsening respiratory disease, pulmonary hypertension, or other complications of extreme prematurity. Sudden cardiorespiratory deterioration, increasing ventilatory requirements, bradycardia, hypotension, poor peripheral perfusion, metabolic acidosis, or unexplained cardiomegaly should therefore prompt consideration of pericardial effusion in an infant with central venous access [2,4]. Bedside echocardiography is particularly valuable in this setting because it enables rapid confirmation of pericardial fluid, assessment of chamber compression and tamponade physiology, and prompt transition to definitive management [3,4].

The pathogenesis of catheter-associated pericardial effusion is multifactorial and may involve catheter-related myocardial or endocardial injury, catheter tip position and subsequent migration, and the characteristics of infused solutions [1-3]. Importantly, pericardial effusion may develop even when catheter position initially appears satisfactory, emphasizing that initial radiographic confirmation does not eliminate the risk of subsequent catheter-related complications [2,3]. Detailed mechanisms, however, may not be clinically demonstrable in an individual patient, and continued vigilance throughout the catheter dwell period remains essential.

Prompt recognition and intervention are critical because established cardiac tamponade can rapidly compromise cardiac output and progress to cardiovascular collapse. Emergency pericardiocentesis, together with removal or repositioning of the implicated catheter, is the cornerstone of management when hemodynamic compromise is present [1,3,4]. Preventive strategies include meticulous catheter placement, appropriate confirmation of catheter tip position, avoidance of intracardiac tip positioning, secure fixation, and continued surveillance for catheter migration [2,4,5].

Despite increasing recognition of catheter-associated pericardial effusion, massive effusions requiring emergency pericardiocentesis in infants at the limits of viability remain exceptionally uncommon. Published reports involving infants weighing less than 700 g are particularly limited, leaving relatively little clinical experience to guide recognition and emergency management in this population. The technical challenges of performing pericardiocentesis in an extremely small and physiologically unstable infant further compound the difficulty.

We report the case of a 24-week gestation male infant weighing 640 g, who developed massive pericardial effusion with cardiac tamponade during the third week of life in the setting of central venous access. The infant underwent emergency pericardiocentesis with drainage of approximately 13 mL of pericardial fluid, followed by rapid cardiorespiratory stabilization and subsequent survival with reassuring neurodevelopmental follow-up. This case is noteworthy not only because of the infant’s extreme prematurity and ELBW, but also because of the extraordinary magnitude of the effusion relative to the infant’s size and the successful emergency intervention at the limits of viability. It highlights the importance of maintaining a high index of suspicion, obtaining prompt bedside echocardiography, and proceeding rapidly to definitive intervention when an ELBW infant with central venous access develops unexplained cardiorespiratory deterioration.

## Case presentation

We report a rare case of massive pericardial effusion causing hemodynamic compromise in a 24-week gestation ELBW neonate weighing 640 g, who required emergency pericardiocentesis during neonatal intensive care unit stay. Despite the extreme prematurity and critical illness, the infant survived with a favorable short-term neurodevelopmental outcome, highlighting the importance of early recognition, bedside echocardiography, and timely intervention in this potentially fatal complication.

The male neonate was delivered at 24 weeks of gestation to a 29-year-old primigravida mother via emergency lower-segment cesarean section performed for preterm labor with fetal compromise. The infant had a birth weight of 640 g, consistent with ELBW. Maternal antenatal history was significant for hypothyroidism. A single dose of antenatal corticosteroid was administered before delivery.

At birth, the neonate exhibited severe respiratory insufficiency with poor respiratory effort, necessitating immediate endotracheal intubation and invasive mechanical ventilation. The infant was subsequently admitted to the neonatal intensive care unit for ongoing intensive cardiorespiratory support. The neonatal course was complicated by severe respiratory distress syndrome requiring surfactant therapy and ventilatory support, persistent pulmonary hypertension of the newborn (PPHN), hemodynamic instability necessitating vasoactive support, and necrotizing enterocolitis managed conservatively. Owing to prolonged dependence on parenteral nutrition, vasoactive medications, and intravenous therapy, a PICC was placed.

**Figure 1:**
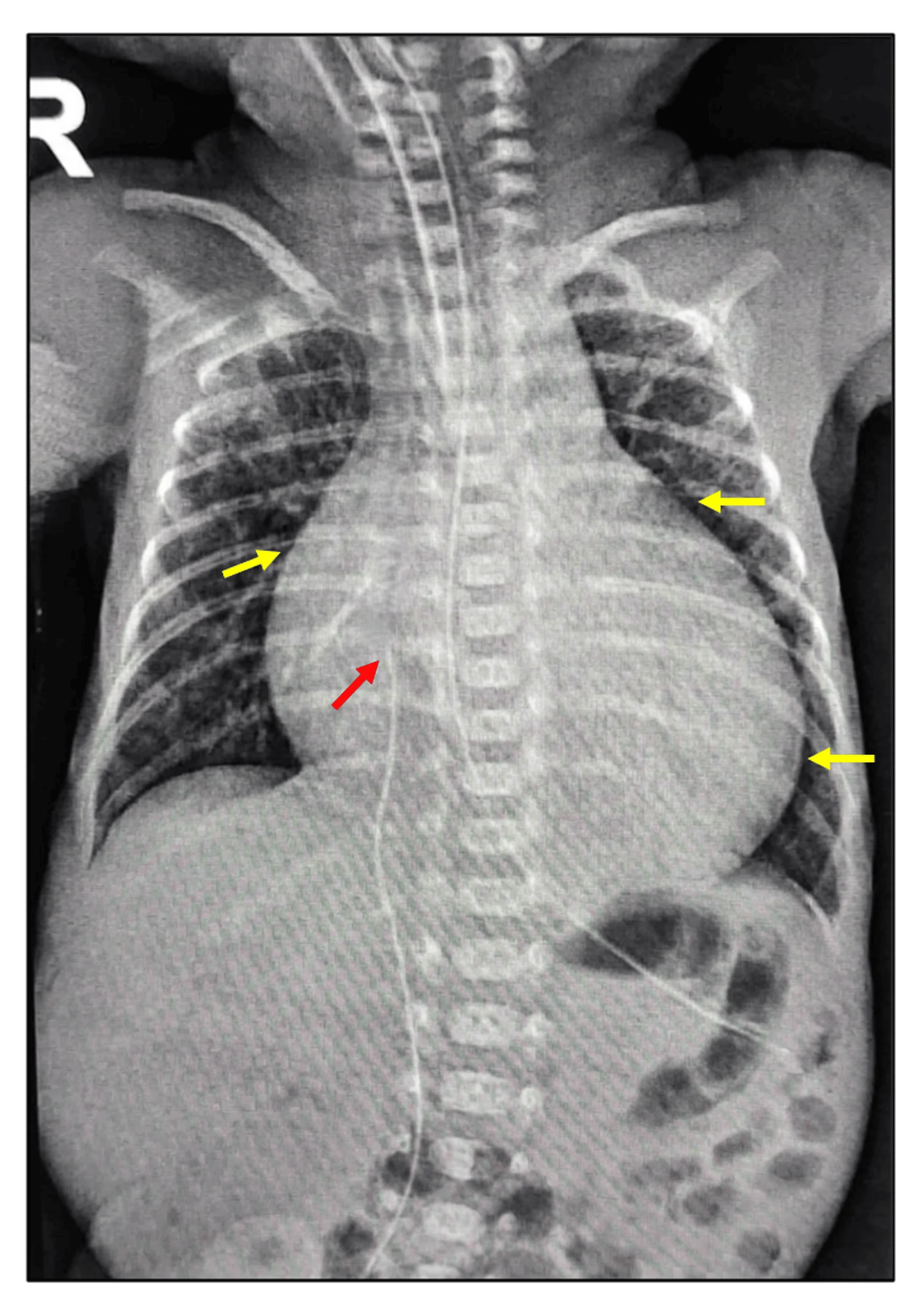
Anteroposterior chest radiograph demonstrating marked cardiomegaly with a characteristic "water-bottle" cardiac silhouette, highly suggestive of a massive pericardial effusion (yellow arrows). The UVC tip projects at the T9 vertebral level, below the diaphragm, indicating a low-lying catheter position (red arrow). UVC: Umbilical venous catheter

During the third week of hospitalization (day of life 21), the infant developed clinical deterioration characterized by escalating ventilatory requirements, recurrent oxygen desaturations, worsening metabolic instability, and progressive hemodynamic compromise. Despite the escalation of respiratory and circulatory support, the infant continued to deteriorate clinically. Chest radiography demonstrated marked cardiomegaly with a “water-bottle” cardiac silhouette suggestive of massive pericardial effusion (Figure 1).

The PICC tip was noted in proximity to the cardiac silhouette, raising a strong suspicion of catheter-associated pericardial effusion with impending cardiac tamponade. In view of the radiographic findings and acute cardiorespiratory decompensation, the PICC was immediately removed.

**Figure 2:**
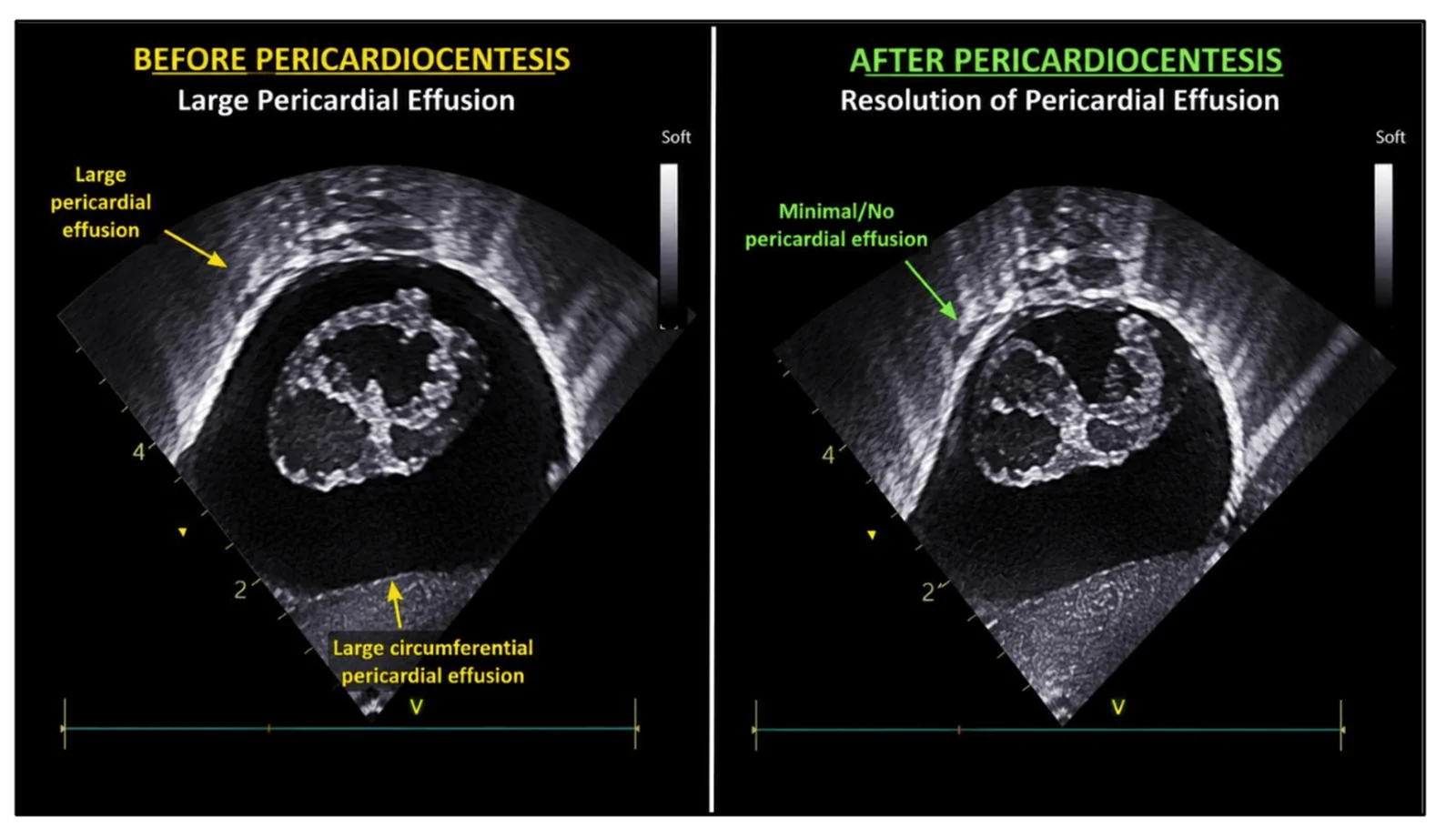
Apical four-chamber echocardiographic images before and after pericardiocentesis showing a large circumferential pericardial effusion with tamponade physiology (left) and near-complete resolution of the effusion following drainage (right)

Echocardiography confirmed the presence of massive pericardial effusion with significant compression of the cardiac chambers and evidence of cardiorespiratory compromise consistent with evolving tamponade physiology (Figure 2).

Emergency pericardiocentesis was performed, and approximately 13 mL of pericardial fluid was aspirated. Performing invasive pericardial drainage in a 640 g infant at the threshold of viability posed substantial technical and physiological challenges. Extreme prematurity, minimal pericardial reserve volume, severe ventilator dependency, fragile hemodynamic status, and the limited margin for procedural error increased the complexity and risk associated with intervention. Nevertheless, the procedure was completed successfully without immediate procedural complications. Following pericardial drainage, the infant demonstrated cardiorespiratory stabilization with reduction in ventilatory requirements, improved lung expansion, enhanced systemic perfusion, and normalization of cardiovascular parameters. Pericardial fluid was aspirated and sent for microbiological culture. Routine biochemical analysis, including glucose, protein, and triglyceride estimation, was not performed because emergency pericardiocentesis was undertaken as a life-saving intervention. Therefore, the precise biochemical composition of the pericardial fluid could not be determined. The pericardial fluid culture showed no growth. Serial echocardiographic evaluations performed demonstrated complete resolution of the pericardial effusion without evidence of re-accumulation.

The remainder of the neonatal intensive care unit stay required prolonged respiratory support, antimicrobial therapy, blood product transfusions, and multidisciplinary intensive care. The infant gradually achieved cardiopulmonary stabilization and adequate growth and was discharged successfully after a prolonged hospitalization. Follow-up demonstrated satisfactory catch-up growth and reassuring neurodevelopmental progress without significant hearing or visual, or major neurodevelopmental impairment.

## Discussion

Massive pericardial effusion with cardiac tamponade is an uncommon but potentially catastrophic complication in neonates and represents one of the most feared adverse events associated with central venous catheterization [5,6]. Although catheter-related pericardial effusion has been increasingly recognized with the widespread use of UVCs and PICCs, reports in ELBW infants remain exceedingly rare. The present case is particularly noteworthy because successful emergency pericardiocentesis was performed in a 24-week gestation infant weighing only 640 g, representing a neonate at the threshold of viability. Published experience with pericardiocentesis in infants weighing less than 700 g is extremely limited, making this case an important addition to the existing literature [5,7].

The increasing survival of extremely premature infants has been accompanied by greater utilization of long-term central venous access for administration of parenteral nutrition, vasoactive medications, and prolonged intensive care support. These devices are indispensable in neonatal intensive care units; they are associated with several potentially serious complications, including infection, thrombosis, pleural effusion, myocardial perforation, and cardiac tamponade [4]. Among these, pericardial effusion remains particularly concerning because clinical deterioration is often abrupt, nonspecific, and rapidly progressive [8].

The reported incidence of catheter-related pericardial effusion in neonates ranges from 0.07% to 2%, although the true incidence is likely underestimated because some cases present as sudden cardiovascular collapse or are diagnosed only at postmortem examination [2,5]. The largest available systematic review and meta-analysis reported a pooled incidence of 3.8 per 1000 central venous catheters and an overall mortality rate of approximately 27% [5]. Importantly, nearly 92% of affected infants developed overt cardiac tamponade, highlighting the aggressive nature of this complication. Furthermore, mortality was significantly higher among infants who did not undergo prompt drainage, emphasizing that early recognition and pericardiocentesis remain the most important determinants of survival [5,6]. Similar observations were reported by Nowlen et al., who demonstrated mortality rates approaching 75% in untreated tamponade compared with substantially improved survival following emergency pericardiocentesis [1]. Prematurity and low birth weight appear to be major risk factors for catheter-related tamponade. In the systematic review, the median birth weight of affected infants was approximately 1,180 g, with the majority of cases occurring in preterm neonates [5]. The rarity of reported cases in infants below 700 g likely reflects both the low incidence of the condition and the substantial technical challenges associated with diagnosis and intervention in infants born at the limits of viability. The susceptibility of ELBW infants is biologically plausible because the neonatal myocardium is structurally immature, with reduced contractile elements, increased water content, an underdeveloped sarcoplasmic reticulum, and markedly thinner atrial walls. In addition, portions of the neonatal atrial wall may contain only endocardium and epicardium with minimal intervening myocardium, rendering them particularly vulnerable to catheter-induced trauma [5,9]. These anatomical features are even more pronounced in infants born at 24-25 weeks gestation and may explain why relatively minor catheter-related trauma can result in myocardial injury and subsequent pericardial effusion. The pathogenesis is therefore multifactorial, with mechanical irritation of the atrial or myocardial wall by the catheter tip considered the most widely accepted mechanism. Repetitive contact may result in endothelial injury, inflammation, myocardial necrosis, erosion, and eventual perforation [10,11]. Hyperosmolar total parenteral nutrition (TPN), calcium-containing solutions, and lipid emulsions may further exacerbate tissue injury through osmotic and chemical effects [6]. Experimental and pathological studies have demonstrated myocardial edema, endothelial disruption, and vascular channel dilatation in affected infants, while histopathological evidence of atrial wall injury and interstitial edema has been reported even in the absence of obvious perforation. These findings suggest that leakage of infusate into the pericardial space may occur through microscopic myocardial damage, a hypothesis further supported by reports in which aspirated pericardial fluid closely resembled infused TPN solution [7,8].

Catheter migration is another contributor to delayed tamponade. Although intracardiac catheter tip position is a recognized risk factor, tamponade may still occur despite apparently satisfactory initial placement [9,10]. More than 60% of reported cases of PICC-related pericardial effusion are associated with intracardiac catheter migration, while catheter angulation, coiling, and myocardial impingement are also recognized mechanisms [11]. Catheter tip position may change over time due to limb movement, postnatal weight loss, alterations in thoracic dimensions, respiratory support, and umbilical stump contraction. These factors are particularly relevant in very low birth weight infants, whose shorter superior vena cava length increases the risk of catheter migration into the cardiac silhouette despite initially appropriate placement [4,5,11]. Consequently, tamponade may develop several days after uncomplicated catheter placement. In our patient, delayed clinical deterioration despite prior catheter stability likely reflected progressive migration and ongoing myocardial injury.

One of the greatest challenges in managing neonatal tamponade is its nonspecific clinical presentation. Reported manifestations include sudden oxygen desaturation, apnea, bradycardia, tachycardia, hypotension, poor perfusion, worsening ventilatory requirements, metabolic acidosis, cyanosis, pallor, and cardiovascular collapse [10]. Cardiac arrest occurs in more than one-fifth of reported cases, with death often occurring within 24 hours of symptom onset. Most affected neonates present with sudden cardiovascular collapse, while others develop progressive cardiorespiratory instability, often preceded by unexplained tachycardia. These findings highlight the narrow window available for diagnosis and timely intervention [5,9].

Chest radiography may provide important diagnostic clues, including progressive cardiomegaly and a globular (“water-bottle”) cardiac silhouette [12]. However, these findings may be subtle in ELBW infants, as even small volumes of pericardial fluid can produce tamponade physiology. In addition, radiography has limited accuracy in defining catheter tip position relative to the pericardial reflection. Increasing evidence supports ultrasound-guided catheter tip confirmation and serial surveillance, particularly in ELBW infants, to facilitate early detection of catheter migration and prevent life-threatening complications [13]. Real-time ultrasound guidance during PICC insertion has been shown to improve catheter tip localization and reduce the incidence of tip malposition, thereby potentially decreasing catheter-related complications [14].

Echocardiography is the diagnostic gold standard for pericardial effusion and cardiac tamponade [15]. It allows rapid bedside identification of pericardial fluid, cardiac chamber collapse, and tamponade physiology, enabling prompt diagnosis in unstable neonates [16]. In our patient, echocardiography rapidly confirmed massive pericardial effusion and guided emergency pericardiocentesis, underscoring its critical role in neonatal intensive care.

The most compelling aspect of the present case is the successful performance of pericardiocentesis in a 640 g infant born at 24 weeks’ gestation. Performing pericardiocentesis at the threshold of viability presents unique challenges because of the extremely small thoracic cavity, narrow pericardial space, cardiorespiratory instability, and limited procedural margin for error. Potential complications include myocardial perforation, coronary artery injury, arrhythmias, pneumothorax, hemothorax, and catastrophic hemorrhage [17,18]. Consequently, reports of successful emergency pericardiocentesis in infants weighing less than 700 g remain exceptionally rare.

The volume of pericardial fluid evacuated in this infant is notable in relation to his extremely low body weight. Approximately 13 mL of fluid was drained from a 640 g neonate. Assuming an estimated circulating blood volume of approximately 50 mL, this volume represents nearly one-quarter of the infant’s estimated circulating blood volume. Although this comparison is illustrative rather than physiologically equivalent, it underscores the substantial pericardial fluid burden relative to the size of the infant. In a 24-week neonate, characterized by small cardiac chambers and limited cardiovascular reserve, accumulation of this magnitude would be expected to significantly impair ventricular filling and cardiac output. The successful emergency evacuation of 13 mL, with subsequent rapid hemodynamic improvement, highlights the severity of tamponade and the potential for prompt pericardial decompression to reverse otherwise life-threatening cardiovascular compromise in an infant at the limits of viability.

Previous reports have shown that even small volumes of pericardial fluid can cause profound hemodynamic compromise in ELBW infants. One of the smallest reported survivors was a 25 weeks, 620 g infant with PICC-associated tamponade, in whom aspiration of only 2 mL of pericardial fluid resulted in immediate cardiovascular stabilization [7]. Similar rapid recovery following emergency drainage has been described in other ELBW and very low birth weight infants [8,19]. These observations, together with the present case, highlight the limited intrapericardial reserve volume and extreme preload dependence of ELBW neonates, in whom relatively small effusions may significantly impair cardiac output.

The literature consistently demonstrates the survival benefit of prompt pericardiocentesis. Neonates undergoing drainage procedures have substantially lower mortality than those managed conservatively, and delayed diagnosis remains a major contributor to adverse outcomes [5]. Early recognition, rapid echocardiographic confirmation, and immediate drainage are therefore critical components of management.

An additional noteworthy feature of our case is the favorable short-term neurodevelopmental outcome despite profound prematurity and severe hemodynamic instability. Long-term outcome data following neonatal tamponade remain limited because many affected infants do not survive the acute event. Delayed diagnosis may result in prolonged cerebral hypoperfusion, hypoxic-ischemic injury, and multiorgan dysfunction. In contrast, our patient demonstrated satisfactory growth and reassuring neurodevelopmental progress during follow-up, emphasizing the importance of timely intervention before irreversible organ injury occurs.

Prevention remains the most effective strategy for reducing catheter-related morbidity and mortality. Recommended measures include meticulous catheter insertion, confirmation of tip position using ultrasonography or echocardiography whenever feasible, avoidance of intracardiac catheter placement, secure catheter fixation, routine surveillance for migration, and periodic reassessment of catheter function [10,20]. Catheter tips should ideally remain outside the cardiac silhouette and pericardial reflection to minimize the risk of myocardial injury [20]. Any unexplained cardiorespiratory deterioration in a neonate with a central venous catheter should prompt immediate reassessment of catheter position and evaluation for pericardial effusion.

In conclusion, catheter-related massive pericardial effusion with cardiac tamponade is a rare but potentially fatal complication in ELBW infants. Early suspicion, prompt echocardiographic diagnosis, and emergency pericardiocentesis are essential for survival. This case adds to the limited literature demonstrating successful pericardiocentesis in infants weighing less than 700 g and underscores the importance of vigilance, preventive catheter practices, and rapid multidisciplinary intervention in improving outcomes at the limits of viability.

## Conclusions

Prevention remains the most effective strategy for reducing catheter-related morbidity and mortality. Recommended measures include meticulous catheter insertion, confirmation of tip position using ultrasonography or echocardiography whenever feasible, avoidance of intracardiac catheter placement, secure catheter fixation, routine surveillance for migration, and periodic reassessment of catheter function. Catheter tips should ideally remain outside the cardiac silhouette and pericardial reflection to minimize the risk of myocardial injury. Any unexplained cardiorespiratory deterioration in a neonate with a central venous catheter should prompt immediate reassessment of catheter position and evaluation for pericardial effusion.

In conclusion, catheter-related massive pericardial effusion with cardiac tamponade is a rare but potentially fatal complication in ELBW infants. Early suspicion, prompt echocardiographic diagnosis, and emergency pericardiocentesis are essential for survival. This case adds to the limited literature demonstrating successful pericardiocentesis in infants weighing less than 700 g and underscores the importance of vigilance, preventive catheter practices, and rapid multidisciplinary intervention in improving outcomes at the limits of viability.
